# IDO1 Inhibitor RY103 Suppresses Trp-GCN2-Mediated Angiogenesis and Counters Immunosuppression in Glioblastoma

**DOI:** 10.3390/pharmaceutics16070870

**Published:** 2024-06-28

**Authors:** Zikang Xing, Xuewen Li, Zhen Ning Tony He, Xin Fang, Heng Liang, Chunxiang Kuang, Aiying Li, Qing Yang

**Affiliations:** 1 State Key Laboratory of Genetic Engineering, School of Life Sciences, MOE Engineering Research Center of Gene Technology, Shanghai Engineering Research Center of Industrial Microorganisms, Fudan University, Songhu Road 2005, Shanghai 200438, China; 17110700017@fudan.edu.cn (Z.X.); 22210700119@m.fudan.edu.cn (X.L.); 21210700126@m.fudan.edu.cn (Z.N.T.H.); 18110700076@fudan.edu.cn (X.F.); 19110700091@fudan.edu.cn (H.L.); 2 Shanghai Key Lab of Chemical Assessment and Sustainability, School of Chemical Science and Engineering, Tongji University, Siping Road 1239, Shanghai 200092, China; kuangcx@tongji.edu.cn; 3 Helmholtz International Lab for Anti-Infectives, Shandong University-Helmholtz Institute of Biotechnology, State Key Laboratory of Microbial Technology, Shandong University, Qingdao 266237, China; ayli@sdu.edu.cn

**Keywords:** glioma, angiogenesis, indoleamine 2,3-dioxygenase 1, tryptophan depletion, general control nonderepressible 2

## Abstract

Glioma is characterized by strong immunosuppression and excessive angiogenesis. Based on existing reports, it can be speculated that the resistance to anti-angiogenic drug vascular endothelial growth factor A (VEGFA) antibody correlates to the induction of novel immune checkpoint indoleamine 2,3-dioxygenase 1 (IDO1), while IDO1 has also been suggested to be related to tumor angiogenesis. Herein, we aim to clarify the potential role of IDO1 in glioma angiogenesis and the mechanism behind it. Bioinformatic analyses showed that the expressions of IDO1 and angiogenesis markers VEGFA and CD34 were positively correlated and increased with pathological grade in glioma. IDO1-overexpression-derived-tryptophan depletion activated the general control nonderepressible 2 (GCN2) pathway and upregulated VEGFA in glioma cells. The tube formation ability of angiogenesis model cells could be inhibited by IDO1 inhibitors and influenced by the activity and expression of IDO1 in condition medium. A significant increase in serum VEGFA concentration and tumor CD34 expression was observed in IDO1-overexpressing GL261 subcutaneous glioma-bearing mice. IDO1 inhibitor RY103 showed positive anti-tumor efficacy, including the anti-angiogenesis effect and upregulation of natural killer cells in GL261 glioma-bearing mice. As expected, the combination of RY103 and anti-angiogenesis agent sunitinib was proved to be a better therapeutic strategy than either monotherapy.

## 1. Introduction

Gliomas are the most common primary brain tumors. Glioblastoma (GBM, World Health Organization (WHO) grade IV glioma) accounts for ~50% of glioma cases and is one of the most refractory tumors with the highest mortality rate in the world [1]. High immune suppression is one of the pathological features of glioma [1,2,3]. The reverse of immunosuppression and the initiation of anti-tumor immunity holds great promise in glioma treatment. Natural Killer cell (NK cell) therapy, CAR-T cell therapy and immune checkpoint inhibition have been proposed as promising treatment strategies for glioma [2]. Additional studies searching for new immunosuppressive components in glioma tumor microenvironments (TME) and exploring their potential as targets for glioma treatment are crucial.

Indoleamine 2,3-dioxygenase 1 (IDO1) catalyzes the first and rate-limiting step in the kynurenine (Kyn) pathway (KP) of tryptophan (Trp) catabolism, which plays an important role in immunity [4]. IDO1-mediated Trp depletion results in the proliferation arrest and anergy induction of T cells through activation of the general control nonderepressible 2 (GCN2) pathway and inhibition of the mammalian target of the rapamycin (mTOR) pathway. IDO1-mediated Kyn accumulation activates the aryl hydrocarbon receptor (AhR) pathway, promoting the differentiation of naive T cells into regulatory T (Treg) cells, and proliferation of other immunosuppressive cells, including myeloid-derived suppressor cells (MDSC), thus restraining anti-tumor immune responses [4,5,6,7]. IDO1 has been recognized as one of the most studied immune checkpoints [8]. It has been indicated that high IDO1 expression is linked with a poor outcome in GBM patients, and an IDO1 blockade with an IDO1 inhibitor Epacadostat (INCB024360) or Indoximod achieves good therapeutic efficacy in glioma-bearing mice [9,10]. Tryptanthrin derivative RY103 is an inhibitor that can blockade KP efficiently and retard tumor growth [11,12]. The combination strategy of RY103 with other therapies against glioma is worth exploring.

Glioma growth and invasion are heavily reliant on angiogenesis, and an increase in vascular density is considered a major feature of glioma malignancy [13,14]. Vascular density in GBM is significantly higher than in lower-grade gliomas, and the increase in vascular density is strongly associated with poor prognosis [15]. The high expression of vascular density marker CD34 in gliomas is associated with high-grade gliomas, leading to its application as a potential diagnostic and prognostic marker for glioma patients [16]. The vascular endothelial growth factor (VEGF) protein family is the most important and potent pro-angiogenic factor, with its most widely expressed and most important family member, VEGFA, playing a crucial role in GBM angiogenesis [14,17,18]. The need for anti-angiogenic therapies has led to the creation of antibodies against VEGFA and its receptor VEGFR and small molecule tyrosine kinase inhibitors (TKIs) with affinity for all VEGFR subtypes such as sunitinib or sorafenib [19,20,21]. However, high consumption of anti-angiogenic agents can lead to the acquisition of drug resistance and tumor immune escape [22,23]. Recent studies suggest that the acquired drug resistance may be related to immunosuppression and increased IDO1 expression [24,25,26]. Given that IDO1 can induce immunosuppressive immune cells, including Treg cells in TME, and anti-VEGF therapy correlates to the increase in IDO1 expression, it is worth exploring whether the addition of IDO1 inhibitors improves the performance of anti-angiogenic drugs against glioma.

IDO1 has been suggested to be associated with angiogenesis, which may also play a role in drug resistance. Studies using human umbilical vein endothelial cells (HUVECs), angiogenesis model cells, treated with media supernatant of fibroblasts, or Lewis lung carcinoma (LLC) cells, have shown that the tube-forming ability of HUVEC could be affected by the expression and activity of IDO1 in media supernatant [27,28,29]. Other in vivo studies have shown that IDO1 expression contributes to tumor angiogenesis in ovarian cancer and lung adenocarcinomas [30,31]. Previous reports also claim that the activation of GCN2 and subsequent nuclear entry of activating transcription factor 4 (ATF4) could play a role in tumor angiogenesis [32,33]. Therefore, it would be interesting to investigate whether IDO1 could deplete Trp, thereby activating the GCN2 pathway, which can be detected using C/EBP homologous protein (CHOP) as a marker [5], and upregulating VEGFA expression in glioma.

In this study, we investigate the mechanism by which IDO1 induces angiogenesis in glioma and aim to provide new evidence supporting dual IDO1 and VEGFA blockade as a viable anti-tumor therapeutic strategy.

## 2. Materials and Methods

### 2.1. Patient Samples

Thirteen newly diagnosed glioma patients were classified into grade I/II, n = 3; grade III/IV, n = 10, according to the WHO standard at Xinhua Hospital from January 2015 to December 2017. The clinical characteristics of the 13 glioma and 5 non-glioma patients are shown in Supplementary Table S1. The study was approved by the Human Ethics Committee of Fudan University and Xinhua Hospital and was conducted in accordance with the Declaration of Helsinki. Informed consent was obtained from all patients.

### 2.2. Animals

Female, 6-week-old, C57BL/6 mice were purchased from Shanghai Jiesijie Experiment Animal Co., Ltd. (Shanghai, China) The experimental procedures were approved by the Animal Ethics Committee of Fudan University and performed in compliance with ARRIVE guidelines.

### 2.3. Cell Culture

Mouse glioma cell line GL261 (RRID:CVCL_Y003), human glioma cell lines U87MG (RRID:CVCL_0022), U251 (RRID:CVCL_0021), A172 (RRID:CVCL_0131) and human embryonic kidney epithelial cell line 293T (RRID:CVCL_0063) were obtained from the Shanghai Cell Bank of Chinese Academy of Sciences. HUVEC cell line (RRID:CVCL_2959) was obtained from the American Type Culture Collection, and hCMEC/D3 cell line (RRID:CVCL_U985) was purchased from MilliporeSigma (Burlington, VT, USA). Cell lines were authenticated by the respective manufacturers by STR profiling. GL261, U87MG, U251, A172, 293T, hCMEC/D3 cells were cultured in Dulbecco’s Modified Eagle Medium (DMEM, Gibco, Thermo Fisher Scientific, Waltham, MA, USA, Cat# 12800017) supplemented with 10% Fetal Bovine Serum (FBS, Capricorn Scientific, Ebsdorfergrund, Germany, Cat# FBS-12A), 100 U/mL penicillin (Aladdin, Shanghai, China, Cat# S432673) and 100 μg/mL streptomycin (Aladdin, Cat# S432673). HUVEC cells were cultured in an endothelial cell culture medium (ScienCell, Carlsbad, CA, USA, Cat# A105484). Cells were grown in a humidified incubator at 37 °C under 5% CO_2,_ and regularly checked for mycoplasma contamination. Since the therapeutic efficacy of various agents was to be investigated in a mouse model, GL261 was mainly examined in the study.

### 2.4. Reagents

HPBCD ((2-hydroxypropyl)-*β*-cyclodextrin, Aladdin, Cat# H108813), 1-MT (1-methyl-L-Tryptophan, Sigma-Aldrich, Saint Louis, MI, USA, Cat# 447439), sunitinib (APExBIO, Cat# A8255), INCB (INCB024360, Selleck, Houston, TX, USA, Cat# S7910), TMZ (temozolomide, Sigma-Aldrich, Cat# 76899) were purchased from respective manufacturers. RY103 (purity > 99%) was designed and synthesized by our lab (Supplementary Table S2, part of the data was adopted from our previous reports [11,12]). These compounds were dissolved in corresponding culture media for in vitro experiments or 10% HPBCD (%*w*/*v*, in 0.9% NaCl solution) for in vivo experiments.

### 2.5. Immunohistochemistry

Immunohistochemistry analysis was performed as reported previously [11]. Samples were incubated by anti-IDO1 (1:500, Proteintech, Rosemont, IL, USA, Cat# 66528–1-Ig), anti-VEGFA (1:100, ABclonal, Woburn, MA, USA, Cat# A21647), anti-CD34 (1:100, ABclonal, Cat# A22197) followed by goat anti-rabbit secondary antibody (1:200, Servicebio, Wuhan, China, Cat# GB23303) or goat anti-mouse secondary antibody (1:200, Servicebio, Cat# GB23301). The slides were then developed by using a 3,3-diaminobenzidine solution (Servicebio, Cat# G1212) and counterstained with hematoxylin (Servicebio, Cat# G1004) and examined under a light microscope.

### 2.6. Cell Transfections

Plasmids (pcDNA3.1(+) empty vector and pcDNA3.1(+)-mIDO1 vector) were transfected into GL261 cells using a Lip2000 transfection reagent (Biosharp, Labgic, Beijing, China, Cat# BL623B) following the manufacturer’s protocol. After transfections, GL261 cells were cultured for 24 h or 72 h.

### 2.7. High-Performance Liquid Chromatography (HPLC) Analysis of Trp and Kyn

HPLC was performed as reported previously [12].

### 2.8. RT-PCR and Quantitative Real-Time PCR

Total RNA was extracted from glioma cells or tumor tissues of mice using Trizol reagent (Servicebio, Cat# G3013). RT-PCR was performed to synthesize cDNA using Premium One-Step RT-PCR kit (ABclonal, Cat# RK20429). Real-time PCR was performed in triplicate for the detection of IDO1, CHOP, VEGFA, CD34, MMP2, MMP9, VEGFR2, CD105, CD31, Factor VIII mRNA expression using the SYBR Green PCR Master Mix kit (ABclonal, Cat# RK21203). β-actin was used as an internal control. The amplification program was set according to our previous report [12]. The primers used for the real-time PCR are shown in Supplementary Table S3.

### 2.9. Western Blot (WB)

WB analyses were performed as described previously [12]. Reagent and antibodies used are listed as follows: RIPA lysis buffer (Beyotime, Shanghai, China, Cat# P0013B), BCA Protein Assay Reagent (Beyotime, Cat# P0009), ECL reagents (Tanon, Shanghai, China, Cat# 1805001), anti-ATF4 (1:1000, Bioss, Cat# bs-1531R), anti-IDO1 (1:3000, Proteintech, Cat# 66528–1-Ig), anti-VEGFA (1:1000, ABclonal, Cat# A21647), anti-CHOP (1:500, ABclonal, Cat# A20987), anti-p-GCN2 (1:500, Bioss, Woburn, MA, USA, Cat# bs-3155R), anti-GCN2 (1:500, ABclonal, Cat# A2307), anti-CD34 (1:500, ABclonal, Cat# A22197), anti-β-actin (1:5000, ABclonal, Cat# AC004), HRP-conjugated anti-mouse secondary antibody (Epizyme, Cambridge, MA, USA, Cat# LF101) and anti-rabbit secondary antibody (Epizyme, Cat# LF102).

### 2.10. Cell Counting Kit-8 (CCK-8) Assay

The viabilities of cells were evaluated by CCK-8 assay (Servicebio, Cat# G4103). The cell medium was replaced with 100 μL fresh medium containing 10 μL CCK-8 solution, and cells were incubated at 5% CO_2_ in a humidified incubator at 37 °C for 1–4 h. The absorbance at a wavelength of 450 nm was detected by a microplate reader (Thermo Fisher Scientific, Waltham, MA, USA).

### 2.11. IDO1 Activity Assay

Cell-based IDO1 activity assay was performed according to reference [34]. Kyn signal was detected and recorded by measuring absorbance at 492 nm by a microplate reader (Thermo Fisher Scientific, Waltham, MA, USA). Cellular IC_50_ values were determined via nonlinear regression analysis using Prism 6 software (Version 6.07, GraphPad Software, La Jolla, CA, USA).

### 2.12. Cell Clone Formation Assay

GL261 cells were digested by 0.25% trypsin solution at the logarithmic phase and then delivered into 12-well culture plates with 500 cells added to each well. The medium was refreshed every 3 days until cell clones could be observed with the naked eye. After 7 days, clones were fixed in carbinol for 30 min and stained with 1 mL crystal violet for 1 h, then analyzed by Image J software (Version 1.53t, U. S. National Institutes of Health, Bethesda, MD, USA).

### 2.13. Tube Formation Assay

Matrigel (Biocoat, Corning Inc., Corning, NY, USA, Cat# 356230) was diluted in DMEM without FBS (DMEM:Matrigel = 1:1). In each well of a 96-well or 24-well culture plate, the plates were covered with 50 μL or 200 μL of Matrigel in DMEM and cultured for 1 h at 37 °C in 5% CO_2_. HUVECs or hCMEC/D3 cells (5 × 10^5^ cells/mL) were resuspended in DMEM with no FBS and seeded on Matrigel. For the 96-well plate, 20,000 cells were added per well, while for the 24-well plate, 150,000 cells per well. After the cells had adhered to Matrigel, the culture medium was discarded, and the cells were cultured in DMEM or prepared conditioned medium. After further incubation for 6 hours, images were acquired under a microscope.

Conditional media were defined as the media supernatants from cells endogenously expressing IDO1, over-expressing IDO1, over-expressing IDO1 supplemented with IDO1 inhibitor, mixed with fresh DMEM at the ratio of 1:1. Supernatants were collected from the following groups before being mixed with DMEM. Control: transfected with pcDNA3.1 empty plasmid and incubated for 72 h; IDO1-OE: transfected with pcDNA3.1-mIDO1 plasmid and incubated for 72 h; IDO1-OE+RY103: transfected with pcDNA3.1-mIDO1 plasmid and incubated with 100 nM RY103 for 72 h; IDO1-OE+1-MT group: transfected with pcDNA3.1-mIDO1 plasmid and incubated with 100 μM 1-MT for 72 h.

### 2.14. Retroviral Infection

Plasmids (VSVG vector, GAG vector and pBABE-mIDO1 vector) were transfected into 293T cells using Lip2000 transfection reagent following the manufacturer’s protocol, and retroviruses carrying pBABE-mIDO1 vectors were produced. Retroviral supernatant of 293T cells was harvested 48 h after initial plasmid transfection and was used to infect GL261 cells. After 48 h, GL261 cells were selected with 10 ng/L puromycin (Aladdin, Cat# P432998) for 5 days to obtain a GL261 cell line stably overexpressing IDO.

### 2.15. Cell Treatment Conditions

GL261 cells were exposed to the following conditions: control, transfected with empty plasmid pcDNA3.1(+) and incubated for 24 h or 72 h; IDO1-OE, transfected with pcDNA3.1(+)-mIDO1 plasmid and incubated for 24 h or 72 h; Trp+, incubated with DMEM without FBS containing 78 μM Trp for 8 h or 24 h; Trp-, incubated with DMEM without FBS or Trp for 8 h or 24 h; RY103, incubated with 0.1–10 μM RY103 for 24 h; IDO1-OE+RY103, transfected with pcDNA3.1(+)-mIDO1 plasmid and then incubated with 100 nM or 1 μM RY103 for 24 h; IDO1-OE+1-MT, transfected with pcDNA3.1(+)-mIDO1 plasmid and then incubated with 100 μM 1-MT for 24 h.

U87MG, U251 and A172 cells were exposed to the following conditions: Trp+, incubated with DMEM without FBS containing 78 μM Trp for 8 h or 24 h; Trp-, incubated with DMEM without FBS or Trp for 8 h or 24 h.

HUVECs and hCMEC/D3 cells were exposed to the following conditions: control, incubated with DMEM without FBS for 6 h; Sunitinib, incubated with DMEM without FBS containing 100 nM sunitinib for 6 h; RY103, incubated with DMEM without FBS containing 100 nM RY103 for 6 h; 1-MT, incubated with DMEM without FBS containing 100 μM 1-MT for 6 h.

### 2.16. Enzyme-Linked Immunosorbent Assay (ELISA)

The concentrations of VEGFA in the medium and serum were measured by ELISA kit (Fuyuanbio, Shanghai, China, Cat# CK-E20738) following the manufacturer’s protocols.

### 2.17. Glioma Allograft Mice Model and Treatment

GL261 subcutaneous glioma-bearing mice: GL261 cells or IDO1-overexpressing GL261 cells were subcutaneously injected into the right forelimb armpit with 2 × 10^6^ cells per mouse. Six days after the implantation, the mice were randomized and divided into groups for different treatments. Treatments were initiated on the same day. Reagents were dissolved in 10% HPBCD (%*w*/*v*, in 0.9% NaCl solution). Control, RY103, 1-MT group mice received 10% HPBCD, 6 mg/kg of RY103 and 100 mg/kg 1-L-MT, respectively, every 36 h, intraperitoneally. Sunitinib group mice were orally administered with 40 mg/kg sunitinib every day for 5 consecutive days. According to the above methods, RY103+Sunitinib group mice were treated with both RY103 and sunitinib, and 1-MT+Sunitinib group mice were treated with both 1-MT and sunitinib. The perpendicular diameters of the tumors were measured every 2 days using Vernier scale calipers, and the tumor volume was calculated using the formula: tumor size = long diameter × (short diameter)^2^/2. The mice were sacrificed after fourteen days of treatment. The spleen weight, tumor weight, and body weight were recorded.

GL261 orthotopic glioma-bearing mice: Tumor implantation was performed as described previously [11]. Six days after the implantation, the mice were randomized and divided into groups. Treatments were initiated on the same day. Reagents were dissolved in 10% HPBCD. Control, RY103, 1-MT, INCB group mice received 10% HPBCD, 6 mg/kg of RY103, 100 mg/kg 1-L-MT, 6 mg/kg INCB024360, respectively, every 36 h, intraperitoneally. TMZ group mice were intraperitoneally administered with 6 mg/kg TMZ every day for 5 consecutive days. RY103+ TMZ group mice were treated with both RY103 and TMZ according to the above methods. The tumor volumes were measured by magnetic resonance imaging (MRI). The mice were sacrificed after twenty-one days of treatment.

### 2.18. Immunofluorescence

Immunofluroscence was performed similarly to the immunohistochemistry process described previously [12]. The following primary antibodies were used: anti-IDO1 and anti-CD34. The following secondary antibodies were used: Alexa Fluor 488-conjugated goat-anti-rabbit (1:200, Servicebio, Cat# GB25303), Cy3-conjugated goat-anti-mouse (1:200, Servicebio, Cat# GB21301). The cell nuclei were then stained with DAPI (1:1000, Beyotime, Cat# C1002). The cell slices were imaged using a laser scanning confocal microscope (Olympus, Tokyo, Japan).

### 2.19. MRI

MRI was performed and analyzed as previously described [11].

### 2.20. Flow Cytometry (FCM) Analysis

Cells from tumors and spleens of mice were exposed to appropriate fluorescence-conjugated antibodies for 30 min at 4 °C in the dark, then washed and resuspended in PBS containing 1% fetal bovine serum. The following antibodies were used:

FITC anti-mouse CD45 (Biolegend, San Diego, CA, USA, Cat# 103108), APC anti-mouse CD3 (Biolegend, Cat# 100236), PE/Cyanine7 anti-mouse CD4 (Biolegend, Cat# 100422), PE anti-mouse CD8 (Biolegend, Cat# 100708), PE-conjugated anti-mouse CD49b (BD Pharmingen, BD Biosciences, Franklin Lakes, NJ, USA, Cat# 558759) and APC-Cy7-conjugated anti-mouse CD3e (BD Pharmingen, Cat# 561042), Brilliant Violet 421 anti-mouse CD45 (Biolegend, Cat# 103134), FITC anti-mouse CD11b (Biolegend, Cat# 101206), APC anti-mouse Ly6G (Biolegend, Cat# 127614), PE anti-mouse Ly6C (Biolegend, Cat# 128008). Zombie NIR Fixable Viability Kit (Biolegend, Cat# 423105) and DRAQ5 (UElandy, Suzhou, China, Cat# D4068) were used to assess the live-versus-dead status of cells. Data were acquired using Beckman Coulter’s Gallios flow cytometer (Beckman Coulter, Brea, CA, USA) and analyzed using FlowJo software (Version 10.10.0, FlowJo, LLC, Ashland, OR, USA).

### 2.21. Statistics

All experiments were repeated at least three times as biological repeats. Prism 6 software (Version 6.07, GraphPad Software) was used to create graphs and perform statistical analyses. Data are expressed as the means ± SD. One-way analysis of variance (ANOVA) followed by Dunnett’s post hoc test was used to compare several treatment groups with one control group. Student’s *t*-test was used to determine the difference between two groups. Significance values were set at * *p* < 0.05, ** *p* < 0.01, *** *p* < 0.001 and **** *p* < 0.0001.

The histochemistry score (H score) for immunohistochemistry slides was obtained as previously described [35].

## 3. Results

### 3.1. The Expression of IDO1 Is Positively Correlated with Angiogenesis in Glioma Patients

**Figure 1:**
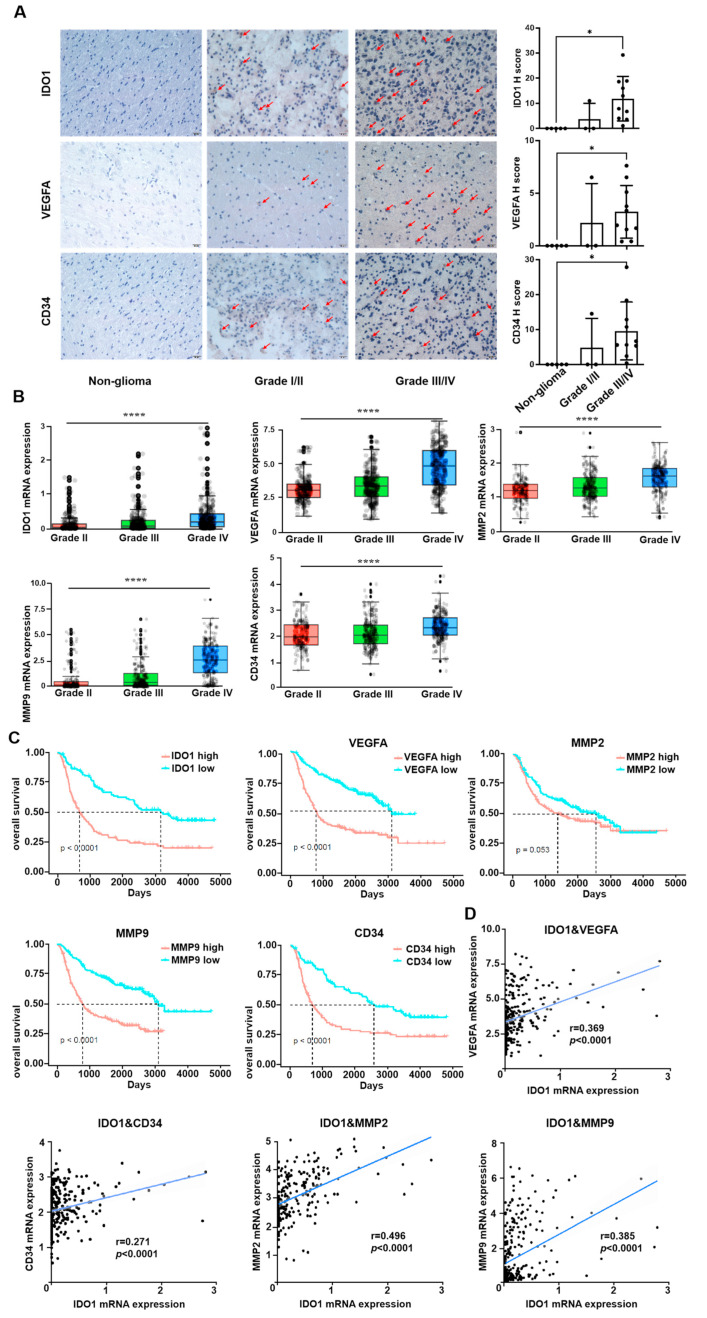
The expression of IDO1, VEGFA, and CD34 in glioma increased with pathological grade, and the expression of IDO1 was positively correlated with the expression of various angiogenic factors and pathological grade, while negatively correlated with the prognosis of glioma patients. Data of B and D are in log_2_(FPKM). (**A**) Expression of IDO1, VEGFA and CD34 in gliomas of different pathological grades. Positive cells are indicated by the arrow. The magnification of the image is 400×, and the length of the scale bar is 20 μm. Non-glioma n = 5, grade I/II n = 3, grade III/IV n = 10. (**B**) The mRNA expression of *IDO1*, *VEGFA*, *MMP2*, *MMP9* and *CD34* in glioma patients at different pathological grades. Data were obtained from the CGGA database, WHO II n = 188, WHO III n = 255, WHO IV n = 249. (**C**) Correlation between the expression of *IDO1, VEGFA*, *MMP2*, *MMP9*, *CD34* and the overall survival of glioma patients. Data were obtained from the CGGA database, Kaplan–Meier curves of overall survival of glioma patients were determined by log-rank test. n = 222 for *IDO1* and *CD34*. n = 404 for *VEGFA*, *MMP2*, *MMP9*. “High” represents the data with higher expression than the median of overall data and “low” with lower expression. (**D**) Correlation between mRNA expressions of IDO1 with *VEGFA*, *CD34*, *MMP2* and *MMP9* in glioma patients. Data were obtained from the CGGA database, n = 404.

Immunohistochemistry staining was employed to determine the expression levels of IDO1, VEGFA, and CD34 in paraffin-embedded tissue sections obtained from 5 non-glioma tissue donors and 13 glioma tissue donors (Supplementary Table S1). In glioma tissue sections, it was observed that the expression levels of IDO1, VEGFA, and CD34 were positively correlated with the pathological grades, with expression levels in grade III/IV glioma patients significantly higher than in patients of lower grade (Figure 1A).

The relationship between IDO1 and angiogenesis marker expression in cancer has not been analyzed by bioinformatics analysis, even though the positive effect of IDO1 expression on angiogenesis in ovarian tumor and lung cancer had been reported [30,31]. We therefore investigated the relationship between IDO1 and angiogenesis-related genes *VEGFA*, *MMP2*, *MMP9*, *CD34*, as well as the association between the mRNA expression levels of these genes and the pathological grade or prognosis of glioma patients, using glioma patient data from the Chinese Glioma Genome Atlas (CGGA [36]). Analysis was performed by built-in functions from the database. High expression levels of these genes were associated with higher grades and poor prognosis of glioma patients (Figure 1B,C). In addition, the expression of IDO1 was weakly yet positively correlated with the expression of angiogenesis-related genes (Figure 1D).

### 3.2. IDO1 Overexpression Derived Trp Deficiency Activates GCN2 Pathway and Upregulates VEGFA Expression

**Figure 2:**
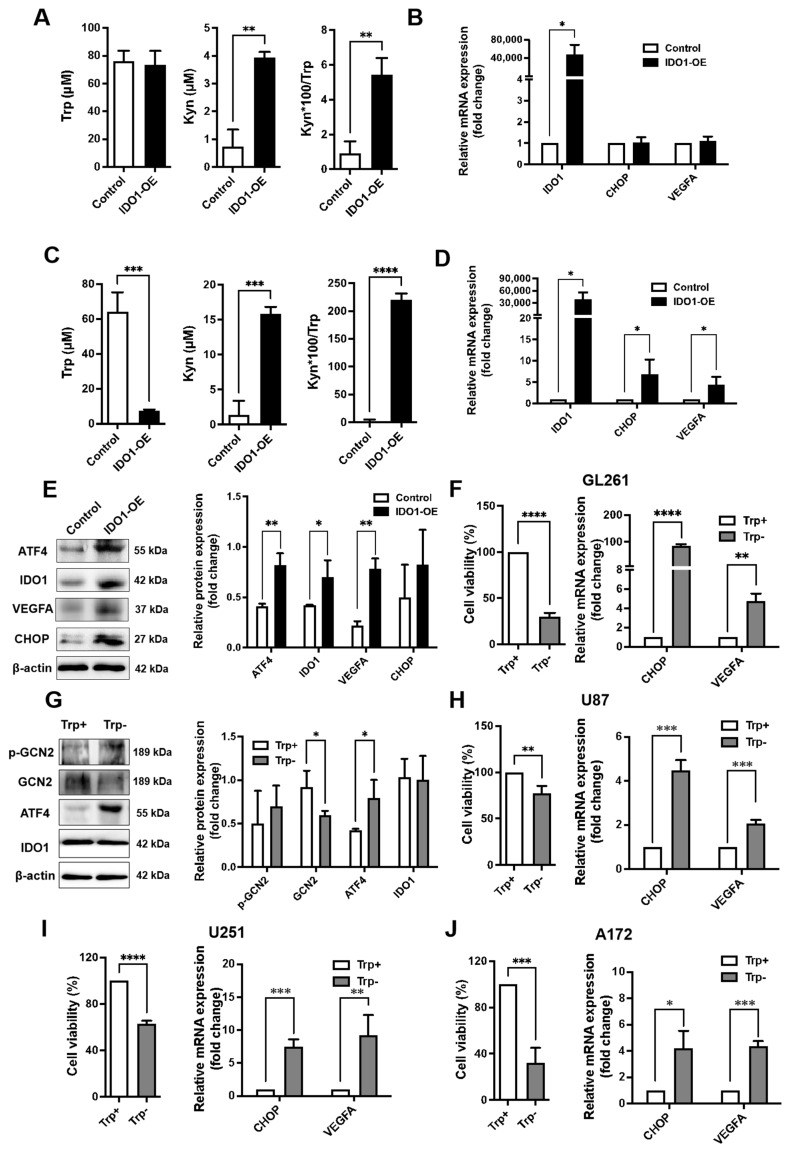
Trp deficiency caused by overexpression of IDO1-activated GCN2 pathway induced the expression of VEGFA. GL261 cells were transfected with IDO1 expressing plasmids and grown in regular medium for 24 h (**A**,**B**) or 72 h (**C**–**E**). Wild-type GL261 (**F**,**G**), U87 (**H**), U251 (**I**), A172 (**J**) cells were grown in Trp-deficient medium for 24 h. The levels of Trp and Kyn were detected by HPLC, and the Kyn/Trp ratio was calculated. Protein and mRNA expression levels were detected by WB and qPCR, respectively, with corresponding expression levels of β-actin serving as internal controls. n = 3/group. (**A**,**C**) Concentration of Trp and Kyn in culture medium. n = 3/group. (**B**,**D**) The mRNA expression levels of *IDO1*, *CHOP*, and *VEGFA*. n = 3/group. E. Protein expression levels of ATF4, IDO1, VEGFA and CHOP. n = 3/group. (**F**,**H**–**J**). The proliferation activity of cells detected by CCK-8, and the mRNA expression levels of *CHOP* and *VEGFA*. n = 3/group. (**G**) Protein expression levels of p-GCN2, GCN2, ATF4 and IDO1. n = 3/group.

We sought to explore if IDO1 modulated glioma angiogenesis via activation of the GCN2 pathway. In IDO1 transiently overexpressing GL261 cells, after being cultured for 24 h, *CHOP* and *VEGFA* mRNA expression levels showed almost no difference compared with the control group (Figure 2B). However, after being cultured for 72 h, CHOP, VEGFA mRNA expression levels as well as ATF4, VEGFA protein expression levels in the cells were significantly increased (Figure 2C–E). As shown in Figure 2A,C, after being cultured for 72 h, the level of Trp depletion in the culture medium of IDO1-overexpressing cells was more drastic than those cultured for 24 h. We speculated that Trp depletion may contribute to the activation of GCN2 and regulation of VEGFA levels. Our hypothesis was partially supported by the lack of significant change in protein expression of GCN2-VEGFA-associated proteins in IDO1 stably overexpressing cells cultured for 72 h, which showed a less drastic decrease in Trp levels (Supplementary Figure S1).

**Figure 3:**
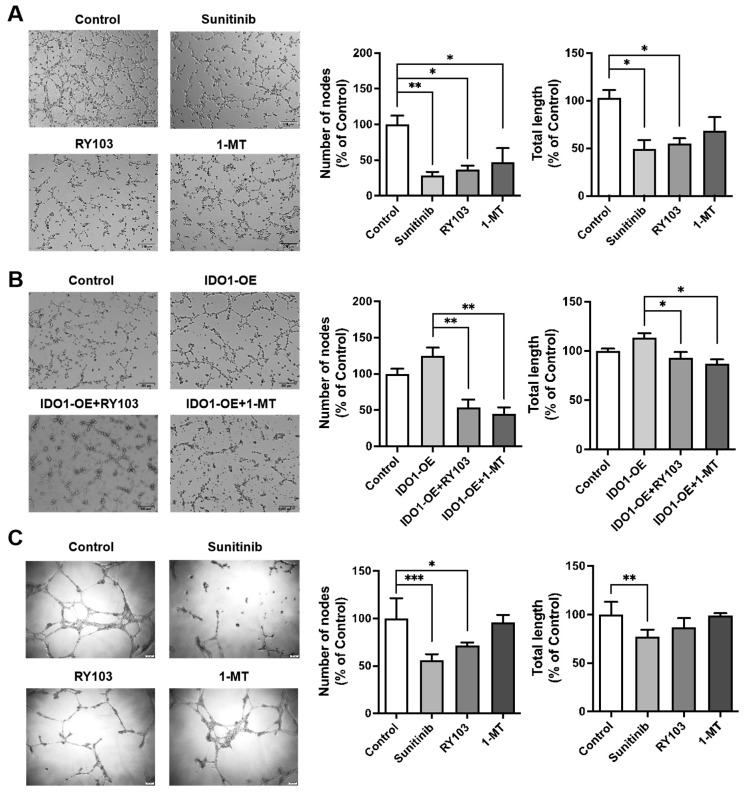
RY103 inhibited the tube-forming ability of hCMEC/D3 and HUVEC cells. The designation of the different treatments is described in the Section 2. Capillary-like structures were detected using a phase-contrast microscope, and the networks formed by hCMEC/D3 cells (**A**,**B**) or HUVEC cells (**C**) were quantified with ImageJ software (Version 1.53t). Magnification, 40 ×; scale bar, 200 µm. n = 3/group. (**A**,**C**) The effect of sunitinib, RY103 and 1-MT on the tube-forming ability of hCMEC/D3 (**A**) or HUVEC (**C**) cells. (**B**) The effect of IDO1 on the tube-forming ability of hCMEC/D3 cells.

The effect of Trp deficiency on the GCN2 pathway and VEGFA expression was then investigated in glioma cell lines GL261, U87MG, U251, and A172. When cultured in Trp-deficient DMEM, proliferative activities of all of these cells were significantly reduced, while *CHOP* and *VEGFA* mRNA expression significantly increased (Figure 2F,H–J). Protein expressions related to the GCN2 pathway in GL261 cells were further analyzed, and the expression level of ATF4 and the phosphorylation of GCN2 were significantly increased (Figure 3G).

These data support the idea that IDO1 overexpression does not directly activate the GCN2 pathway or upregulate VEGFA expression but instead leads to Trp depletion, which activates the GCN2 pathway.

### 3.3. IDO1 Induces the Tube-Forming Ability of HUVECs and hCMEC/D3 Cells

We investigated the respective effect of IDO1 activity and expression on tube-forming ability of HUVECs and hCMEC/D3 cells. Both sunitinib and RY103 significantly inhibited the tube formation of hCMEC/D3 and HUVECs, while 1-MT had a weaker inhibitory effect on hCMEC/D3 cells (Figure 3A,C). Effects of RY103 on cell viability and KP blockage were examined in GL261 before conditional medium treatment on endothelial cells (Supplementary Figure S2). After treating hCMEC/D3 cells with conditioned media from GL261 cells with varying levels of IDO1 expression and activity, we found that overexpression of IDO1 promoted the tube formation of hCMEC/D3 cells, which could be inhibited by RY103 and 1-MT (Figure 3B).

Taken together, we demonstrated that both the activity and expression of IDO1 had impacts on tube-forming ability of HUVECs and hCMEC/D3 cells.

### 3.4. Overexpression of IDO1 Promotes Tumor Angiogenic Factors Expression in GL261 Subcutaneous Glioma-Bearing Mice

**Figure 4:**
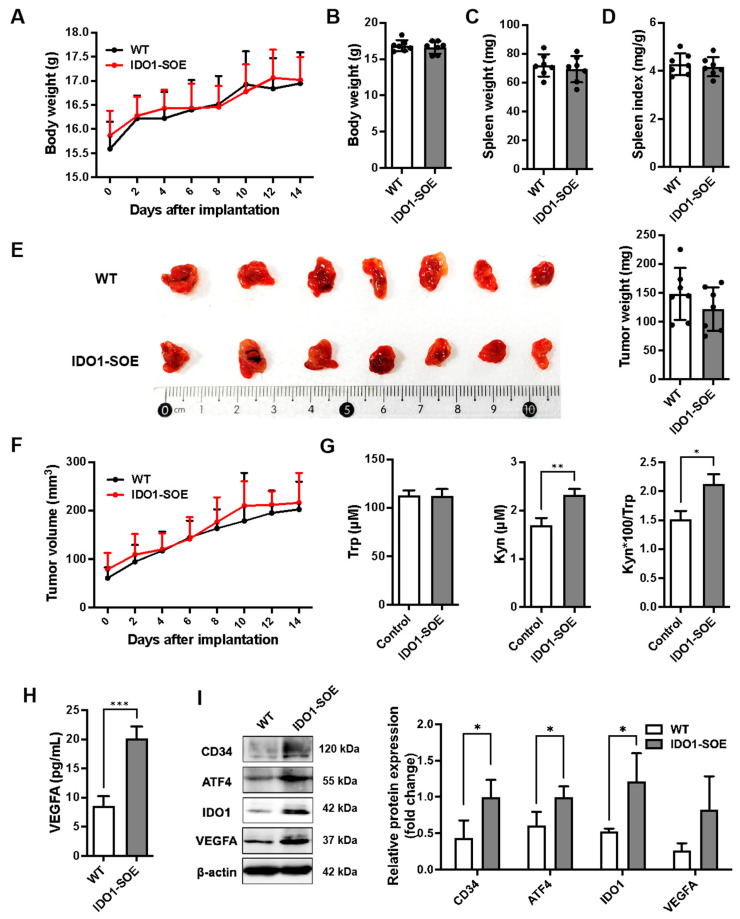
Overexpression of IDO1 promoted expression of angiogenic factors in the tumor of GL261 subcutaneous glioma-bearing mice. The construction of mouse models is described in the Section 2. Mice were sacrificed 14 days post-implantation. The tumors were excised, weighed and photographed. The blood was collected and used for the detection for Trp, Kyn and VEGFA levels. (**A**). Body weight change curve. n = 7/group. (**B**). Body weight before sacrifice. n = 7/group. (**C**) Spleen weight. n = 7/group. (**D**) Splenic index (spleen weight (mg)/body weight (g)). n = 7/group. (**E**). The tumor weight. n = 7/group. (**F**) The tumor volumes. n = 7/group. (**G**) Concentration of Trp and Kyn in serum was detected by HPLC. The Kyn/Trp ratio was also calculated. n = 7/group. (**H**) The concentration of VEGFA in serum was detected by ELISA. n = 7/group. (**I**) Protein expression levels of CD34, ATF4, IDO1 and VEGFA in tumors were detected by WB. β-actin was used as an internal control. n = 3/group.

Mice subcutaneously bearing IDO1 stable overexpressing (IDO1-SOE) and wild-type GL261 subcutaneous glioma-bearing mice (WT) were constructed and compared. The weight change curves, tumor growth curves, weights of the tumors, body weights, spleen weights, and spleen index were not significantly different between the two groups (Figure 4A–F). In IDO1-SOE mice, serum KP activity and VEGFA concentration were increased (Figure 4G,H) accompanied by a rise in the expression of CD34, ATF4, IDO1, and VEGFA in tumor tissues, with the most significant uplift observed in ATF4 and IDO1 expression (Figure 4I).

These results indicated that the overexpression of IDO1 in glioma-bearing mice did not promote tumor growth but activated the GCN2 pathway in tumor tissues, which led to the upregulation of VEGFA. This may be attributed to the function of IDO1 in angiogenesis and malignancy of glioma.

### 3.5. RY103 Downregulates Tumor Angiogenic Factors in GL261 Subcutaneous Glioma-Bearing Mice

**Figure 5:**
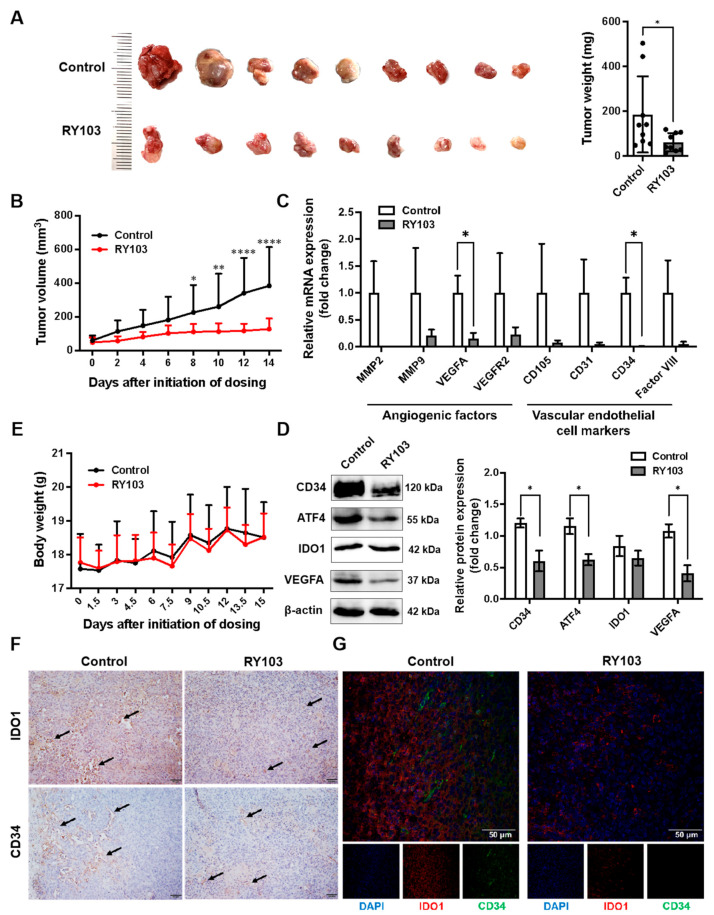
RY103 downregulated angiogenic factors in tumor of GL261 subcutaneous glioma-bearing mice. The construction of mouse models and the designation of the different treatments are described in the Section 2. (**A**) The tumor weight. n = 9/group. (**B**) The tumor volumes. n = 7/group. (**C**) The mRNA expression levels of *MMP2*, *MMP9*, *VEGFA*, *VEGFR2*, *CD105*, *CD31*, *CD34* and Factor VIII in tumors were detected by qPCR. β-actin was used as an internal control. n = 4/group. (**D**) Protein expression levels of CD34, ATF4, IDO1, and VEGFA expression in tumors were detected by WB. β-actin was used as an internal control. n = 3/group. (**E**) Body weight change curve. n = 9/group. (**F**) The expression of IDO1 and CD34 in paraffin sections of tumors was detected by immunohistochemistry. The cells positive for IDO1 or CD34 were indicated by the arrows. The magnification of the image is 200× and the length of the scale is 50 μm. (**G**) The expression of IDO1 and CD34 in tumors was detected by immunofluorescence. The magnification of the image is 400× and the length of the scale is 50 μm.

The effect of the KP blockade by IDO1 inhibitor RY103 on tumor angiogenesis was evaluated with GL261 subcutaneous glioma-bearing mice. The results showed that RY103 significantly inhibited tumor growth and reduced tumor weight (Figure 5A,B) without an obvious effect on body weight (Figure 5E). The results also showed that RY103 significantly reduced the mRNA expression levels of VEGFA and CD34 and the protein levels of CD34, ATF4, and VEGFA (Figure 5C,D), among various angiogenic factors and endothelial markers. Immunohistochemistry and immunofluorescence analyses show that the expressions of IDO1 and CD34 in tumor tissues of RY103-treated mice were reduced (Figure 5F,G).

These results indicated that RY103 had the ability to inhibit tumor growth and the GCN2 pathway, as well as tumor angiogenesis in GL261 subcutaneous glioma-bearing mice.

### 3.6. RY103 Retards Tumor Growth in GL261 Orthotopic Glioma-Bearing Mice, But the Combination of RY103 and TMZ Shows No Synergistic Effect

We compared the therapeutic effects of RY103 with IDO1 selective inhibitor INCB, and TMZ, a standard chemotherapeutic agent for malignant glioma, as well as the combination of RY103 with TMZ in GL261 orthotopic glioma-bearing mice.

**Figure 6:**
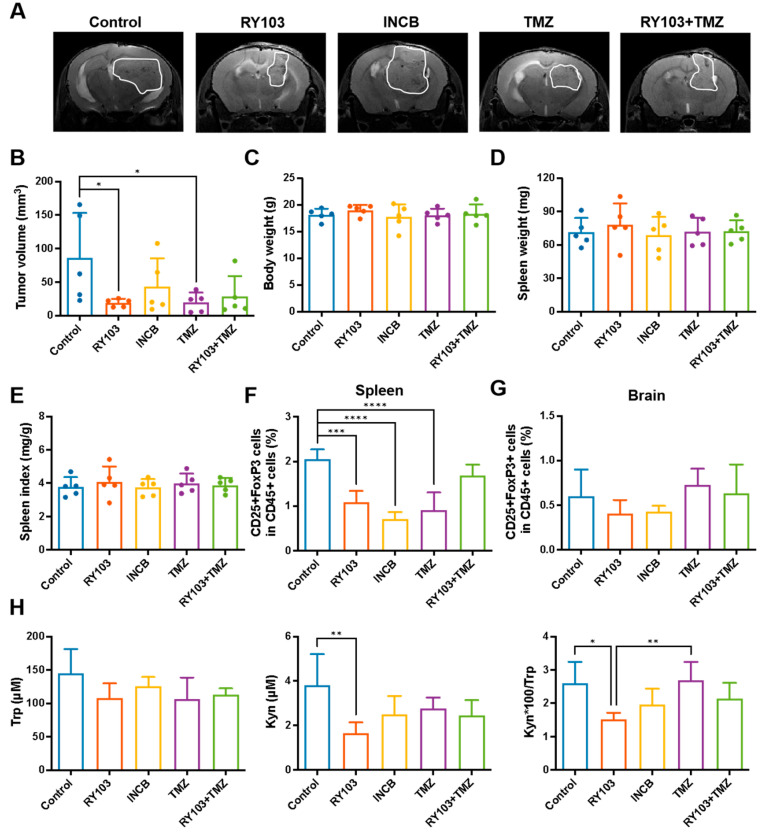
RY103 inhibited tumor growth and decreased Treg proportion in spleens in GL261 orthotopic glioma-bearing mice. The construction of mouse models and the designation of the different treatments are described in the Section 2. (**A**,**B**). The tumor volumes. One day before the mice were sacrificed, the tumor volumes were assessed by MRI. n = 5/group. (**C**) Body weight before sacrifice. n = 5/group. (**D**) Spleen weight. n = 5/group. (**E**) Splenic index (spleen weight (mg)/body weight (g)). n = 5/group. (**F**,**G**). The proportion of Treg cells in the spleens (**F**) or brains (**G**) were detected by FCM. n = 5/group. (**H**) Concentrations of Trp and Kyn in serum were detected by HPLC, and the Kyn/Trp ratio was calculated. n = 5/group.

Among these treatments, RY103 and TMZ significantly inhibited tumor growth but their combination showed no significant effect (Figure 6A,B). The treatments did not affect body weight, spleen weight, and spleen index of the mice (Figure 6C–E). HPLC analysis revealed that RY103 could significantly decrease serum Kyn levels and KP activity in serum (Figure 6H).

FCM analysis revealed that the proportions of Treg cells in the spleens were significantly lower in RY103, INCB and TMZ groups, but the combination treatment showed no significant effect (Figure 6F). In brains, the original site of the tumor, RY103, could also decrease the proportion of Treg cells but with no statistical significance (Figure 6G). In the context of the proportions of CD45+ cells, CD4+ and CD8+ T cells, none of the treatments showed significant effects either in spleens or brains.

In summary, the IDO1 inhibitor RY103 showed good therapeutic efficacy in terms of KP blockage and Treg reduction, suggesting that RY103 had promising therapeutic potential as a treatment for glioma.

### 3.7. The Combo of RY103 and Sunitinib Exhibits Stronger Therapeutic Efficacy than Monotherapy in GL261 Subcutaneous Glioma-Bearing Mice

**Figure 7:**
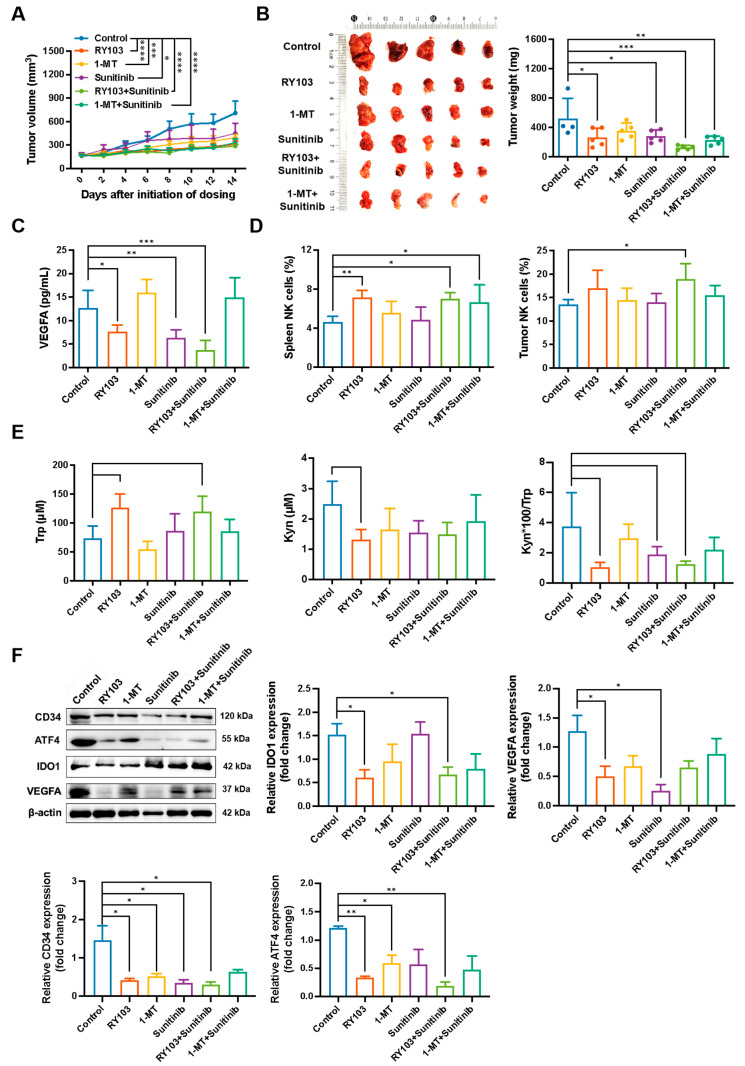
The combination of RY103 and sunitinib exhibited stronger antitumor and anti-angiogenesis effects in GL261 subcutaneous glioma-bearing mice. The construction of mouse models and the designation of the different treatments are described in the Section 2. (**A**) The tumor volumes. n = 5/group. (**B**) The tumor weight. n = 5/group. (**C**) The concentration of VEGFA in serum was detected by ELISA. n = 5/group. (**D**) The proportion of NK cells in the spleens and tumors was detected by FCM. n = 5/group. (**E**) Concentrations of Trp and Kyn in serum were detected by HPLC, and the Kyn/Trp ratio was calculated. n = 5/group. (**F**) Protein expression levels of CD34, ATF4, IDO1 and VEGFA in tumor were detected by WB, β-actin was used as an internal control. n = 3/group.

We then investigated the therapeutic efficacy of the combination of RY103 and sunitinib in GL261 subcutaneous glioma-bearing mice. All treatment options significantly inhibited tumor growth, with the combination of RY103 and sunitinib demonstrating the strongest effect (Figure 7A,B). In addition, both RY103 and its combo with sunitinib significantly upregulated Trp levels, and downregulated Kyn levels and Kyn/Trp ratios in mice serum (Figure 7E). RY103, sunitinib, and their combination significantly downregulated the concentration of VEGFA in serum, with the combo showing the most potent effect (Figure 7C). Western blot analysis revealed that in all treatment groups, CD34 and ATF4 protein expressions were downregulated, with the combination of RY103 and sunitinib demonstrating the most significant reduction. The 1-MT group had higher ATF4 and VEGFA expression than the RY103 group, suggesting that it was less effective in blocking the GCN2 pathway. The combination of RY103 and sunitinib significantly downregulated the protein expressions of CD34 and ATF4, indicating an effective blockage of the GCN2 pathway and thus suppression of tumor angiogenesis (Figure 7F). The ability of RY103 treatment to downregulate angiogenic factors may be a result of its capability not only to block KP but also to upregulate Trp in serum (Figure 7C,E).

NK cells are important immune cells infiltrating the TME of glioma with infiltration scores in low-grade glioma and GBM, sometimes even higher than those of T cells, and NK cell dysfunction plays an important role in immunosuppression in GBM [37,38,39]. Therefore, we detected the proportions of NK cells in spleens and tumor tissues of mice using FCM. The results revealed a significant increase in the proportions of NK cells in the spleens of mice treated with RY103, RY103+Sunitinib, and 1-MT+Sunitinib. In tumors, a significant increase was also observed in the RY103+Sunitinib group (Figure 7D). These results are in line with what we observed in GL261 orthotopic glioma-bearing mice, suggesting RY103 treatment may reverse immunosuppression by upregulating NK cells and downregulating Treg cells (Figure 6F,G and Figure 7D).

Since MDSCs are immunosuppressive cells in the TME of gliomas and high levels of MDSC cells have been associated with higher tumor grades of glioma [40], the proportion of MDSC cells in spleens and tumor tissues are also analyzed by FCM, alongside CD45+, CD4+ and CD8+ T cells. However, none of the treatment groups showed significant effects on these cells compared to the control groups. 

To summarize, our results suggested that the combination of RY103 and sunitinib could significantly inhibit tumor growth, block the GCN2 pathway in tumor tissue, and inhibit tumor angiogenesis. Therefore, the combination of RY103 and sunitinib holds promise as a potential therapeutic approach for the treatment of glioma.

## 4. Discussion

While highly immunosuppressive and robust angiogenic features of glioma have been discovered, therapeutic strategies targeting these features, either still in clinical trials or already commercialized, have shown limited efficacy [2,41]. There is substantial evidence that the activation of novel immune checkpoint IDO1 induces immunosuppression in autoimmune diseases, bacterial and viral infections, and many cancers [42]. IDO1 has been reported to be associated with angiogenesis [30,31,32], but the relationship between IDO1 and angiogenesis in glioma, as well as the mechanisms by which IDO1 regulates angiogenesis, require further investigation.

For the first time, our study examined the association between IDO1 and glioma angiogenesis and the underlying mechanisms. Our result by bioinformatic analysis not only showed the correlation between IDO1 expression and pathological grade of glioma as well as poor prognosis of the patients but also revealed that the expression of IDO1 positively correlates with those of angiogenesis-related genes.

To explore the mechanism behind IDO1 inducing angiogenesis, the study investigated if IDO1 modulated glioma angiogenesis via activation of the GCN2 pathway because of the important role of GCN2 activation in VEGFA upregulation. Previous reports have shown that IDO1 promoted neovascularization by activating GCN2 and inducing IL6 production and that the absence of sulfur-containing amino acids could also trigger GCN2 activation [32,33]. Our study also investigated the role of the GCN2 pathway during angiogenesis, but rather than GCN2 protein itself or other amino acids, we instead focused on the activation of the pathway by Trp depletion, as it was closely affiliated with overexpressed IDO1 in glioma. We found that in glioma cells, IDO1 overexpression only activated the GCN2 pathway and upregulated VEGFA expression if it caused a drastic decrease in Trp levels (Figure 2). Consistent with what we observed in glioma cells, IDO1 overexpression also upregulates VEGFA through GCN2 activation in mice (Figure 4). Based on these results, we first proposed that Trp depletion caused by IDO1 overexpression and KP activation serve as the key to angiogenesis mediated by the GCN2 pathway. Our study also confirmed the positive effect of IDO1 activity and expression on tube formation in vitro with HUVEC and hCMEC/D3 cells (Figure 3). Combining our data, previous in vitro studies using LLC cells [28,29] and in vivo reports [30,31], it is suggested that IDO1-expressing tumor cells could induce the tube formation of adjacent vascular cells, regardless of the cell type or the originating site of the tumor.

Between the two major features of glioma, angiogenesis and immunosuppression, interplay in TME has been reported previously. In glioma, cytokines, chemokines, and growth factors produced by the tumor promote the infiltration of various immune cells into the tissues, although the unique brain immunology leads to a low infiltration ratio, which may explain why we do not observe a significant effect of the treatments on T cell proportions in the brains of orthotopic mice [43,44,45]. Peripheral antitumor immune cells are frequently reprogrammed into a distinct immunosuppressive phenotype in TME soon after their chemotaxis to the tumor, which contributes to promoting tumor development, metastasis, and resistance to cancer therapies [46,47,48]. For example, MDSCs and Tregs are recruited to the tumor site via multiple approaches, both of which further exert an immunosuppressive function mainly through T cell inhibition and apoptosis [49,50]. MDSCs and Tregs also collaborate with tumor-associated macrophages to suppress the activity of NK cells, and the proportions of Tregs and MDSCs are reported to be increased in the glioma TME [51]. In addition, many angiogenic factors have immunosuppressive functions, and high-density vessels in the TME can inhibit immune cell activation [52]. Immune cells can also secrete angiogenesis factors that promote vessel formation. MDSCs can differentiate into phenotypes similar to tumor endothelial cells and integrate into vessels, assisting in creating stable and proliferative vessel walls [53]. Tregs possess the ability to promote blood vessel formation by secreting VEGFA and creating a proangiogenic microenvironment in tumors [54].

As a contributing factor to both angiogenesis and immunosuppression, several studies have shown the importance of IDO1 in glioma [9,11,55,56]. The effect of IDO1 inhibitors on glioma, such as INCB024360, has been drawing attention [10]. Furthermore, it was demonstrated that combining IDO1 inhibition with other immune checkpoint inhibitors could significantly improve therapeutic efficacy [57]. In this study, we investigated the effect of RY103 and its combination with other therapeutic agents on immune cells in TME. Although the combination of RY103 and TMZ had no synergistic effect on glioma and was not an ideal treatment strategy for glioma, it was the first trial on the combination of our novel IDO1 inhibitor and a standard therapeutic agent. The combo of RY103 and sunitinib demonstrated a stronger ability to significantly increase the ratios of NK cells, in both spleens and tumors, inhibit tumor growth and suppress of tumor angiogenesis in subcutaneous glioma-bearing mice (Figure 7D). RY103 treatment was able to upregulate Trp in serum of subcutaneous tumor-bearing mice, counteracting the effect of Trp depletion on angiogenesis. The downregulation of Tregs observed in orthotopic mice, along with the upregulation of NK cells in subcutaneous mice also suggested that RY103 could effectively counter T cell anergy and NK cell inhibition in glioma TME and thus reverse immunosuppression. These results are in line with our previous report that IDO1 inhibitors enhanced NK cell function and immune response [35]. Since NK cells have higher infiltration scores than T cells in GBM [37], indicating their presence and importance in the TME, our findings suggested that RY103 might have great potential as a therapeutic agent against glioma. Our failure to detect significant changes in the proportion of T cells or MDSC indicates that our findings are not perfect. The lack of significant differences in T cells and MDSC proportions among the groups may result from the limited infiltration of these cells into the glioma TME (Figure 6F,G and Figure 7D). Future studies may include investigating the effect of brain infiltration on various immune cells during the treatment of gliomas or GBM, either using RY103 alone or combined with other therapeutic agents.

## 5. Conclusions

In summary, our study demonstrated that IDO1 contributed to glioma angiogenesis by causing Trp depletion in the TME, and IDO1 inhibitor RY103 might be a potential therapeutic choice against glioma. Our research not only enhanced the understanding of the relationship between IDO1 and angiogenesis in glioma in terms of mechanism but also proposed the promising strategy of combining an IDO1 inhibitor with an angiogenesis inhibitor for glioma treatment.

## Data Availability

CGGA data analyzed in this study are publicly available on the CGGA website (http://www.cgga.org.cn/download.jsp, accessed on 27 August 2023) with the DataSet ID being mRNAseq_693. Experimental data generated in this study are available in the article and Supplementary Materials. Further inquiries can be directed to the corresponding author.

## Supplementary Materials

The following supporting information can be downloaded at: https://www.mdpi.com/article/10.3390/pharmaceutics16070870/s1. The manuscript was submitted with the following supplementary files: Supplementary Figure S1: Without drastic Trp depletion, activation of the GCN2 pathway and upregulation of VEGFA expression were not significant. Supplementary Figure S2: RY103 had no cytotoxicity and could significantly inhibit KP activity and clone formation ability of GL261 cells. Supplementary Figure S3: FCM gating strategy for the detection of various T cells. Supplementary Figure S4: FCM gating strategy for the detection of NK (CD3e-CD49b+) cells. Supplementary Table S1: Basic clinical characteristics of tissue donors. Supplementary Table S2: IDO1 and tryptophan-2,3-dioxygenase (TDO) inhibitory activities and chemical structures of 1-L-MT, RY103 and INCB024360. Supplementary Table S3: Primers for quantitative reverse transcription-polymerase chain reaction.

## Abbreviations

1-MT: 1-methyl-tryptophan; AhR: aryl hydrocarbon receptor; ATF4: activating transcription factor 4; CCK-8: cell counting kit-8; CGGA: Chinese Glioma Genome Atlas; CHOP: C/EBP homologous protein; DMEM: Dulbecco’s Modified Eagle Medium; ELISA: enzyme-linked immunosorbent assay; FCM: flow cytometry; FBS: fetal bovine serum; GBM: glioblastoma; GCN2: general control nonderepressible 2; H score: histochemistry score; hCMEC/D3: human cerebral microvascular endothelial cells; HPLC: high-performance liquid chromatography; HUVECs: human umbilical vein endothelial cells; IDO1: indoleamine 2,3-dioxygenase 1; IDO1-OE: IDO1 overexpressing cells/mice; IDO1-SOE: IDO1 stable overexpressing cells/mice; INCB: INCB024360; Kyn: kynurenine; KP: kynurenine pathway; MDSC: myeloid-derived suppressor cells; mTOR: mammalian target of rapamycin; NK cells: natural killer cells; OS: overall survival; PFS: progression-free survival; PD-L1: programmed cell death ligand 1; TDO: tryptophan 2,3-dioxygenase; TKI: tyrosine kinase inhibitors; TME: tumor microenvironment; TMZ: temozolomide; Treg cells: regulatory T cells; Trp: tryptophan; VEGF: vascular endothelial growth factor; VEGFA: vascular endothelial growth factor A; VEGFR: vascular endothelial growth factor receptor.
