# Supplementary material for: IDO1 Inhibitor RY103 Suppresses Trp-GCN2-Mediated Angiogenesis and Counters Immunosuppression in Glioblastoma
Source: Pharmaceutics. 2024 Jun 28;16(7):870. doi: 10.3390/pharmaceutics16070870 (PMC11279595; doi:10.3390/pharmaceutics16070870)
Supplement: Supplementary file 1 [file pharmaceutics-16-00870-s001.zip › pharmaceutics-3032110-supplementary.pdf]

# IDO1 Inhibitor RY103 Suppresses Trp-GCN2-Mediated Angiogenesis and Counters Immunosuppression in Glioblastoma

Zikang Xing [1], Xuewen Li [1], Zhen Ning Tony He [1], Xin Fang [1], Heng Liang [1], Chunxiang Kuang [2], Aiying Li [3] and Qing Yang [1,*]

1 State Key Laboratory of Genetic Engineering, School of Life Sciences, MOE Engineering Research Center of Gene Technology, Shanghai Engineering Research Center of Industrial Microorganisms, Fudan University, Songhu Road 2005, Shanghai 200438, China; 17110700017@fudan.edu.cn (Z.X.); 22210700119@m.fudan.edu.cn (X.L.); 21210700126@m.fudan.edu.cn (Z.N.T.H.); 18110700076@fudan.edu.cn (X.F.); 19110700091@fudan.edu.cn (H.L.)

2 Shanghai Key Lab of Chemical Assessment and Sustainability, School of Chemical Science and Engineering, Tongji University, Siping Road 1239, Shanghai 200092, China; kuangcx@tongji.edu.cn

3 Helmholtz International Lab for Anti-Infectives, Shandong University-Helmholtz Institute of Biotechnology, State Key Laboratory of Microbial Technology, Shandong University, Qingdao 266237, China; ayli@sdu.edu.cn

* Correspondence: yangqing68@fudan.edu.cn; Tel./Fax: +86-21-31240641

# Supplementary Materials

## Supplementary Figures

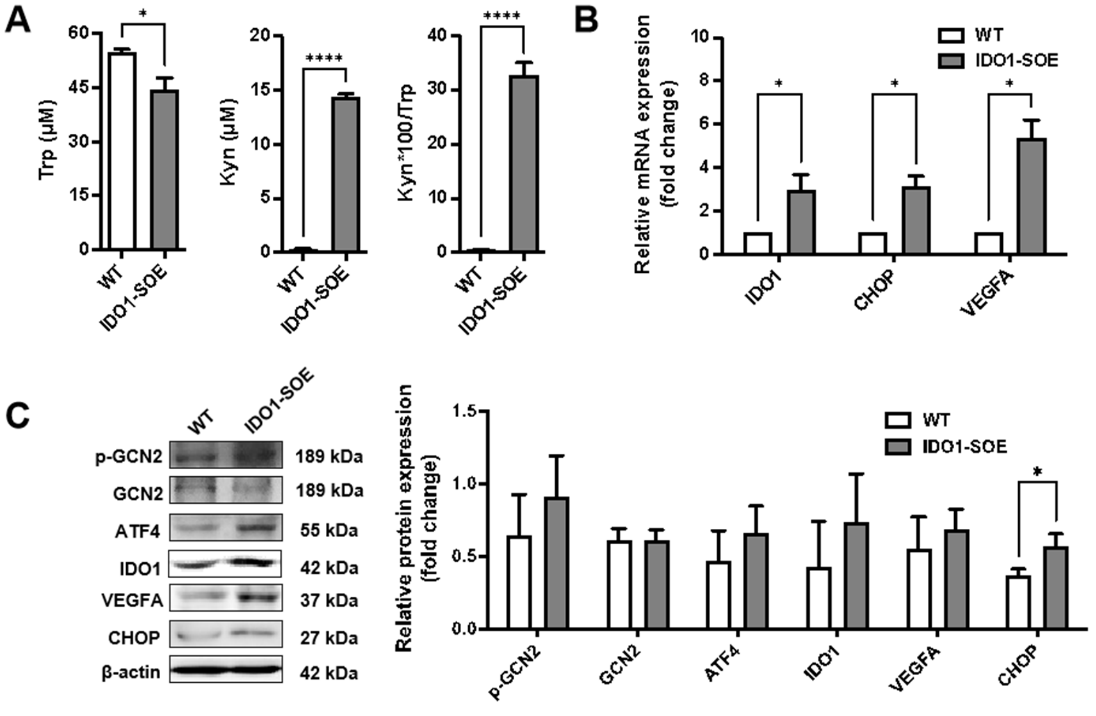


**Supplementary Figure S1. Without drastic Trp depletion, activation of the GCN2 pathway and upregulation of VEGFA expression were not significant.**

IDO1 stable overexpressing GL261 cells (IDO1-SOE) and wild type (WT) GL261 cells were cultured for 72 h. Culture medium, total RNA, and protein were collected. The designation of the different treatments is described in the Materials and Methods section.

A. The levels of Trp and Kyn were detected by HPLC, and the Kyn/Trp ratio was calculated. n=3/group.

B. The mRNA expression levels of IDO1, CHOP, and VEGFA were detected by qPCR. β-actin was used as an internal control. n=3/group.

Protein expression levels of p-GCN2, GCN2, ATF4, IDO1, VEGFA and CHOP were detected by WB. β-actin was used as an internal control. The representative image is shown on the left and the statistical plot is shown on the right. n=3/group.

The data were presented as mean ± SD. Statistical significance was determined by two-tailed unpaired t test (A-C), or one-way ANOVA followed by Dunnett's post hoc test (D-E). * $p<0.05$, ** $p<0.01$, *** $p<0.001$, **** $p<0.0001$.

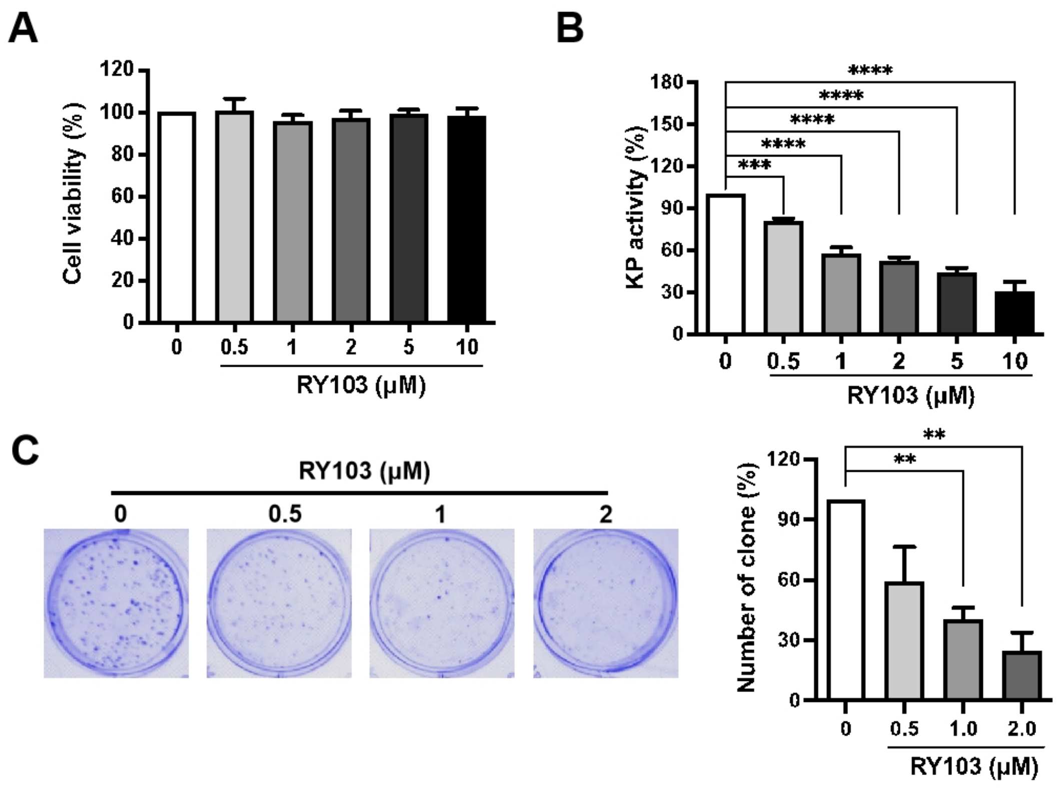


**Supplementary Figure S2. RY103 had no cytotoxicity and could significantly inhibit KP activity and clone formation ability of GL261 cells.**

GL261 cells treated with 0-10 μM RY103 for 24 h (A, B) or with 0-2 μM RY103 for 7 days (C).

A. The viability of the cells was detected by CCK-8. n=3/group.

B. The levels of Trp and Kyn in culture medium were detected by HPLC, and the Kyn/Trp ratio, representing relative KP activity to the 0 μM group, was calculated. n=3/group.

C. The clone formation was assayed. The representative image is shown on the left and statistic plot is presented on the right as percentages relative to the 0 μM group. n=3/group.

The data were presented as mean ± SD. Statistical significance was determined by one-way ANOVA followed by Dunnett's post hoc test. ** $p$<0.01, *** $p$<0.001, **** $p$<0.0001.

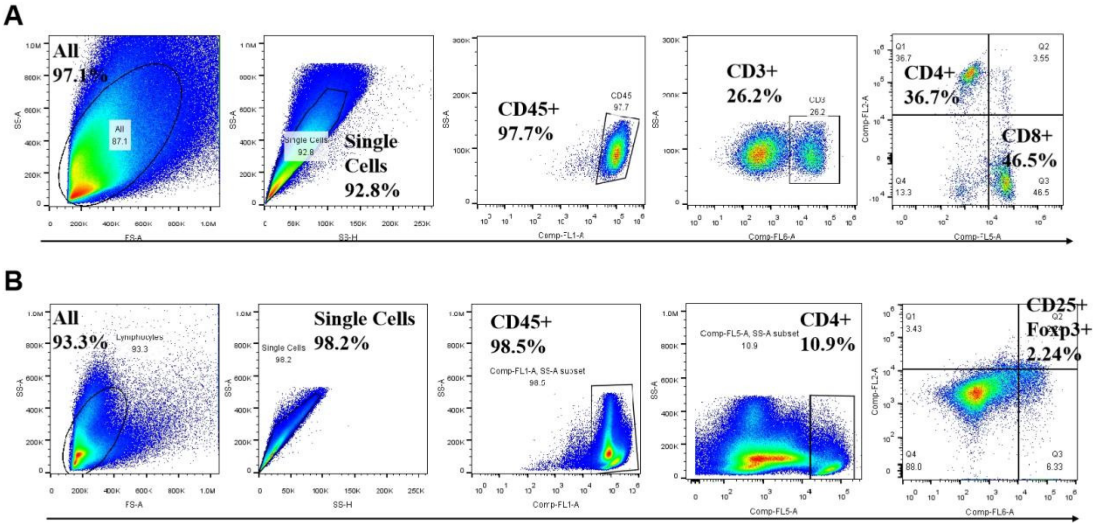


**Supplementary Figure S3. FCM gating strategy for the detection of various T cells.**

A. Gating strategy for detection of CD4+ T cells and CD8+ T cells.

B. Gating strategy for detection of Treg (CD25+FoxP3+) cells.

All gates were set using fluorescence-minus-one (FMO) controls.

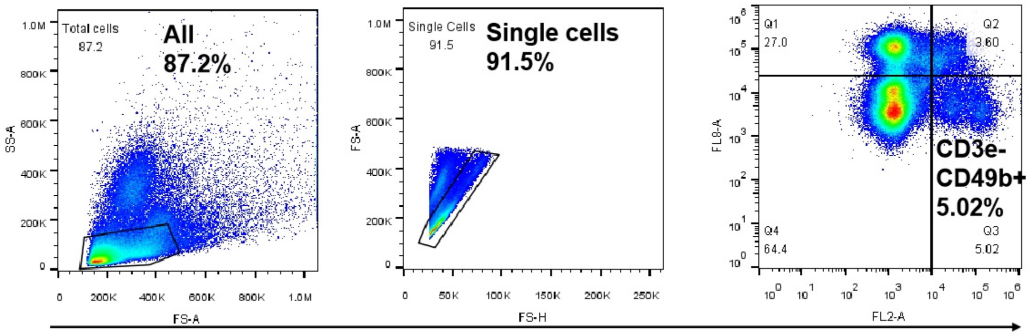


**Supplementary Figure S4. FCM gating strategy for the detection of NK (CD3e-CD49b+) cells.**

All gates were set using fluorescence-minus-one (FMO) controls.

## Supplementary Tables

**Supplementary Table S1. Basic clinical characteristics of tissue donors**

| Variable | Number |
|---|---|
| Non-glioma patients | 5 |
| Pathological grades of glioma patients | |
| grade I/II | 3 |
| grade III/IV | 10 |
| Sex of glioma patients | |
| Male | 7 |
| Female | 6 |
| Age at diagnosis, year | |
| < 50 | 9 |
| ⩾ 50 | 4 |

**Supplementary Table S2. IDO1 and tryptophan-2,3-dioxygenase (TDO) inhibitory activities and chemical structures of 1-L-MT, RY103 and INCB024360.**

| Compound | Chemical Structure | $IC_{50}$ (µM) | | | | Ki (µM) | |
|---|---|---|---|---|---|---|---|
| | | Enzymatic | | Cellular | | | |
| | | IDO1 | TDO | IDO1 | TDO | IDO1 | TDO |
| 1-L-MT | 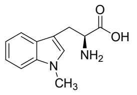 | 380-420 | NI | 17.0-24.0 | ND | 34.0-45.0 | ND |
| RY103 | 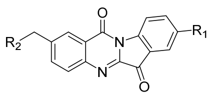 | 1.5-3.0 | 1.5-3.0 | 0.1-0.4 | 0.1-0.4 | 4.5-6.5 | 0.3-0.6 |
| INCB024360 | 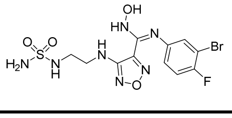 | 0.05-0.09 | NI | 0.007-0.008 | NI | ND | ND |

Ki: Inhibitory constant, $IC_{50}$: Half maximal inhibitory concentration, ND: Not detected, NI: No inhibition, R1 and R2 are kept non-disclosed due to confidentiality reasons.

**Supplementary Table S3. Primers for quantitative reverse transcription-polymerase chain reaction**

| Primer | Sequences (5′-3′) |
|---|---|
| *Human-β-actin* | Forward: CTTCCAGCCTTCCTTCCTGG |
| | Reverse: CCAGGGCAGTGATCTCCTTC |
| *Human-IDO1* | Forward: ATGCAAGAACGGGACACT |
| | Reverse: GCCTTTCCAGCCAGACAA |
| *Human-CHOP* | Forward: TAAAGATGAGCGGGTGGCAG |
| | Reverse: CTGCCATCTCTGCAGTTGGA |
| *Human-VEGFA* | Forward: AGGCCAGCACATAGGAGAGA |
| | Reverse: TACCGGGATTTCTTGCGCTT |
| *Mouse-β-actin* | Forward: CTGTCCCTGTATGCCTCTG |
| | Reverse: ATGTCACGCACGATTTCC |
| *Mouse-IDO1* | Forward: TGTGAATGGTCTGGTCTC |
| | Reverse: CTGTGCCCTGATAGAAGT |
| *Mouse-CD34* | Forward: CAGGAGAAAGGCTGGGTGAAG |
| | Reverse: GTTGTCTTGCTGAATGGCCG |
| *Mouse-CHOP* | Forward: CCTGAGGAGAGAGTGTTCCAG |
| | Reverse: GACACCGTCTCCAAGGTGAA |
| *Mouse-VEGFA* | Forward: AACGATGAAGCCCTGGAGTG |
| | Reverse: GCTGGCTTTGGTGAGGTTTG |
| *Mouse-MMP2* | Forward: AACGGTCGGGAATACAGCAG |
| | Reverse: GTAAACAAGGCTTCATGGGGG |
| *Mouse-MMP9* | Forward: CGACTTTTGTGGTCTTCCCC |
| | Reverse: AGCGGTACAAGTATGCCTCTG |
| *Mouse-VEGFR2* | Forward: TTCACAGTCGGGTTACAGGC |
| | Reverse: CTGCCGACGTTCCTCTCTTT |
| *Mouse-CD105* | Forward: TGGGTATCACCTTTGGTGCC |
| | Reverse: GGAGGCTTGGGATACTCACG |
| *Mouse-CD31* | Forward: GAGCCTCACCAAGAGAACGG |
| | Reverse: ATTGGATGGCTTGGCCTGAA |
| *Mouse-Factor VIII* | Forward: ATGCAAATAGCACTCTTCGCT |
| | Reverse: ACACTGAGCAGATCACTCTGA |
